# Supplementary material for: Evaluation of Rapid Immunochromatographic Tests for the Direct Detection of Extended Spectrum Beta-Lactamases and Carbapenemases in Enterobacterales Isolated from Positive Blood Cultures
Source: Microbiol Spectr. 2021 Dec 8;9(3):e00785-21. doi: 10.1128/Spectrum.00785-21 (PMC8653814; doi:10.1128/Spectrum.00785-21)
Supplement: SUPPLEMENTAL FILE 1 — Supplemental material. Download SPECTRUM00785-21_Supp_1_seq1.pdf, PDF file, 0.3 MB [file spectrum00785-21_supp_1_seq1.pdf]

Supplementary Table 1. Comparison of NG-Test CTX-M MULTI and NG-Test Carba 5 results with reference results

Sample number	Organism Identification by MALDI-TOF	NG-Test CTX-M Multi Results	NG-Test Carba 5 Results	BD Phoenix 100 ESBL Results	BD Phoenix 100 CPO Detect Results	Easyplex SuperBug CRE Results	Cepheid Carba-R PCR Results
Isolates (1-32) were spiked in BD BACTEC bottles to verify the performance of NG-Test CTX-M Multi							
**1.**	*Salmonella typhi*	Positive	ND	Positive	Negative	Positive	ND
**2.**	*Salmonella typhi*	Positive	ND	Positive	Negative	Positive	ND
**3.**	*Escherichia coli*	Positive	ND	Positive	Negative	Positive	ND
**4.**	*Escherichia coli*	Positive	ND	Positive	Negative	Positive	ND
**5.**	*Escherichia coli*	Positive	ND	Positive	Negative	Positive	ND
**6.**	*Escherichia coli*	Positive	ND	Positive	Negative	Positive	ND
**7.**	*Escherichia coli*	Positive	ND	Positive	Negative	Positive	ND
**8.**	*Escherichia coli*	Positive	ND	Positive	Negative	ND	ND
**9.**	*Escherichia coli*	Positive	ND	Positive	Negative	ND	ND
**10.**	*Escherichia coli*	Positive	ND	Positive	Negative	ND	ND
**11.**	*Escherichia coli*	Positive	ND	Positive	Negative	ND	ND
**12.**	*Escherichia coli*	Positive	ND	Positive	Negative	ND	ND
**13.**	*Klebsiella pneumoniae*	Positive	ND	Positive	Negative	Positive	ND
**14.**	*Klebsiella pneumoniae*	Positive	ND	Positive	Negative	Positive	ND
**15.**	*Klebsiella pneumoniae*	Positive	ND	Positive	Negative	Positive	ND
**16.**	*Klebsiella pneumoniae*	Positive	ND	Positive	Negative	Positive	ND
**17.**	*Klebsiella pneumoniae*	Positive	ND	Positive	Negative	Positive	ND
**18.**	*Klebsiella pneumoniae*	Positive	ND	Positive	Negative	ND	ND
**19.**	*Klebsiella pneumoniae*	Positive	ND	Positive	Negative	ND	ND
**20.**	*Klebsiella pneumoniae*	Positive	ND	Positive	Negative	ND	ND
**21.**	*Serratia marcescens*	Negative	ND	Negative	Negative	ND	ND
**22.**	*Serratia marcescens*	Negative	ND	Negative	Negative	ND	ND

Created with
PDFBear.com

**23.**	*Serratia marcescens*	Negative	ND	Negative	Negative	ND	ND
**24.**	*Enterobacter cloacae*	Negative	ND	Negative	Negative	ND	ND
**25.**	*Escherichia coli*	Negative	ND	Negative	Negative	Negative	ND
**26.**	*Escherichia coli*	Negative	ND	Negative	Negative	Negative	ND
**27.**	*Escherichia coli*	Negative	ND	Negative	Negative	Negative	ND
**28.**	*Klebsiella pneumoniae*	Negative	ND	Negative	Negative	Negative	ND
**29.**	*Klebsiella pneumoniae*	Negative	ND	Negative	Negative	Negative	ND
**30.**	*Klebsiella pneumoniae*	Negative	ND	Negative	Negative	Negative	ND
**31.**	*Klebsiella pneumoniae*	Negative	ND	Negative	Negative	Negative	ND
**32.**	*Klebsiella pneumoniae*	Negative	ND	Negative	Negative	ND	ND
Isolates (33-65) were spiked in BD BACTEC bottles to verify the performance of NG-Test Carba 5							
**33.**	*Escherichia coli*	ND	Positive OXA-48-like	Negative	Class D CPO	ND	Positive OXA-48-like
**34.**	*Escherichia coli*	ND	Positive OXA-48-like	Negative	Class D CPO	ND	Positive OXA-48-like
**35.**	*Escherichia coli*	ND	Positive NDM-like	Negative	Class B CPO	ND	Positive NDM-like
**36.**	*Escherichia coli*	ND	Positive NDM-like	Negative	Class B CPO	ND	Positive NDM-like
**37.**	*Escherichia coli*	ND	Positive NDM-like	Negative	Class B CPO	ND	Positive NDM-like
**38.**	*Escherichia coli*	ND	Positive NDM-like	Negative	Class B CPO	ND	Positive NDM-like
**39.**	*Escherichia coli*	ND	Positive IMP-like	Negative	Class B CPO	ND	Positive IMP-like
**40.**	*Escherichia coli*	ND	Positive IMP-like and VIM1-like	Negative	Class B CPO	ND	Positive IMP-like and VIM1-like
**41.**	*Klebsiella pneumoniae*	ND	Positive KPC-like	Negative	Class A CPO	ND	Positive KPC-like
**42.**	*Klebsiella pneumoniae*	ND	Positive VIM1-like	Negative	Class B CPO	ND	Positive VIM1-like
**43.**	*Klebsiella pneumoniae*	ND	Positive OXA-48-like and NDM-like	Negative	CPO Producer	ND	Positive OXA-48-like and NDM-like
**44.**	*Klebsiella pneumoniae*	ND	Positive OXA-48-like and NDM-like	Negative	CPO Producer	ND	Positive OXA-48-like and NDM-like
**45.**	*Klebsiella pneumoniae*	ND	Positive OXA-48-like and NDM-like	Negative	CPO Producer	ND	Positive OXA-48-like and NDM-like

Created with
PDFBear.com

**46.**	*Klebsiella pneumoniae*	ND	Positive OXA-48-like	Negative	Class D CPO	ND	Positive OXA-48-like
**47.**	*Klebsiella pneumoniae*	ND	Positive OXA-48-like	Negative	Class D CPO	ND	Positive OXA-48-like
**48.**	*Klebsiella pneumoniae*	ND	Positive NDM-like	Negative	Class B CPO	ND	Positive NDM-like
**49.**	*Klebsiella pneumoniae*	ND	Positive IMP-like and VIM1-like	Negative	Class B CPO	ND	Positive IMP-like and VIM1-like
**50.**	*Klebsiella pneumoniae*	ND	Negative	Negative	Negative	ND	Negative
**51.**	*Klebsiella pneumoniae*	ND	Negative	Negative	Negative	ND	Negative
**52.**	*Klebsiella pneumoniae*	ND	Negative	Negative	Negative	ND	Negative
**53.**	*Klebsiella pneumoniae*	ND	Negative	Negative	Negative	ND	Negative
**54.**	*Klebsiella pneumoniae*	ND	Negative	Negative	Negative	ND	Negative
**55.**	*Escherichia coli*	ND	Negative	Positive	Negative	ND	Negative
**56.**	*Escherichia coli*	ND	Negative	Positive	Negative	ND	Negative
**57.**	*Escherichia coli*	ND	Negative	Negative	Negative	ND	Negative
**58.**	*Escherichia coli*	ND	Negative	Negative	Negative	ND	Negative
**59.**	*Escherichia coli*	ND	Negative	Negative	Negative	ND	Negative
**60.**	*Escherichia coli*	ND	Negative	Negative	Negative	ND	Negative
**61.**	*Escherichia coli*	ND	Negative	Negative	Negative	ND	Negative
**62.**	*Serratia marcescens*	ND	Negative	Negative	Negative	ND	Negative
**63.**	*Serratia marcescens*	ND	Negative	Negative	Negative	ND	Negative
**64.**	*Serratia marcescens*	ND	Negative	Negative	Negative	ND	Negative
**65.**	*Enterobacter cloacae*	ND	Negative	Negative	Negative	ND	Negative
Isolates (66-114) are positive blood culture that were tested prospectively with both NG CTX-M Multi test and Carba 5 test							
**66.**	*Escherichia coli*	Positive	Negative	Positive	Negative	ND	ND
**67.**	*Escherichia coli*	Positive	Negative	Positive	Negative	ND	ND
**68.**	*Escherichia coli*	Negative	Positive OXA-48-like	Negative	Class D CPO	ND	ND
**69.**	*Escherichia coli*	Negative	Positive NDM-like	Negative	Class B CPO	ND	ND

Created with
PDFBear.com

**70.**	*Escherichia coli*	Negative	Negative	Negative	Negative	ND	ND
**71.**	*Escherichia coli*	Negative	Negative	Negative	Negative	ND	ND
**72.**	*Escherichia coli*	Negative	Negative	Negative	Negative	ND	ND
**73.**	*Escherichia coli*	Negative	Negative	Negative	Negative	ND	ND
**74.**	*Escherichia coli*	Negative	Negative	Negative	Negative	ND	ND
**75.**	*Escherichia coli*	Negative	Negative	Negative	Negative	ND	ND
**76.**	*Escherichia coli*	Negative	Negative	Negative	Negative	ND	ND
**77.**	*Escherichia coli*	Negative	Negative	Negative	Negative	ND	ND
**78.**	*Klebsiella pneumoniae*	Positive	Negative	Negative*	Negative	ND	ND
**79.**	*Klebsiella pneumoniae*	Positive	Negative	Negative*	Negative	ND	ND
**80.**	*Klebsiella pneumoniae*	Positive	Negative	Positive	Negative	ND	ND
**81.**	*Klebsiella pneumoniae*	Negative	Negative	Negative	Negative	ND	ND
**82.**	*Klebsiella pneumoniae*	Negative	Negative	Negative	Negative	ND	ND
**83.**	*Klebsiella pneumoniae*	Negative	Negative	Negative	Negative	ND	ND
**84.**	*Klebsiella pneumoniae*	Negative	Negative	Negative	Negative	ND	ND
**85.**	*Klebsiella pneumoniae*	Negative	Negative	Negative	Negative	ND	ND
**86.**	*Klebsiella pneumoniae*	Negative	Negative	Negative	Negative	ND	ND
**87.**	*Klebsiella pneumoniae*	Negative	Negative	Negative	Negative	ND	ND
**88.**	*Klebsiella pneumoniae*	Negative	Negative	Negative	Negative	ND	ND
**89.**	*Klebsiella pneumoniae*	Negative	Negative	Negative	Negative	ND	ND
**90.**	*Klebsiella pneumoniae*	Negative	Negative	Negative	Negative	ND	ND
**91.**	*Klebsiella pneumoniae*	Negative	Negative	Negative	Negative	ND	ND
**92.**	*Klebsiella pneumoniae*	Negative	Negative	Negative	Negative	ND	ND
**93.**	*Klebsiella pneumoniae*	Negative	Negative	Negative	Negative	ND	ND
**94.**	*Klebsiella pneumoniae*	Negative	Negative	Negative	Negative	ND	ND
**95.**	*Klebsiella pneumoniae*	Negative	Negative	Negative	Negative	ND	ND
**96.**	*Klebsiella pneumoniae*	Negative	Negative	Negative	Negative	ND	ND

Created with
PDFBear.com

**97.**	*Klebsiella pneumoniae*	Negative	Negative	Negative	Negative	ND	ND
**98.**	*Klebsiella pneumoniae*	Negative	Negative	Negative	Negative	ND	ND
**99.**	*Klebsiella oxytoca*	Negative	Negative	Negative	Negative	ND	ND
**100.**	*Klebsiella variicola .*	Negative	Negative	Negative	Negative	ND	ND
**101.**	*Enterobacter cloacae*	Positive	Negative	Negative*	Negative	ND	ND
**102.**	*Enterobacter cloacae*	Negative	Negative	Negative	Negative	ND	ND
**103.**	*Enterobacter cloacae*	Negative	Negative	Negative	Negative	ND	ND
**104.**	*Enterobacter cloacae*	Negative	Negative	Negative	Negative	ND	ND
**105.**	*Enterobacter cloacae*	Negative	Negative	Negative	Negative	ND	ND
**106.**	*Enterobacter cloacae*	Negative	Negative	Negative	Negative	ND	ND
**107.**	*Serratia marcescens*	Negative	Negative	Negative	Negative	ND	ND
**108.**	*Serratia marcescens*	Negative	Negative	Negative	Negative	ND	ND
**109.**	*Serratia marcescens*	Negative	Negative	Negative	Negative	ND	ND
**110.**	*Serratia marcescens*	Negative	Negative	Negative	Negative	ND	ND
**111.**	*Serratia marcescens*	Negative	Negative	Negative	Negative	ND	ND
**112.**	*Serratia marcescens*	Negative	Negative	Negative	Negative	ND	ND
**113.**	*Salmonella spp. not typhi*	Negative	Negative	Negative	Negative	ND	ND
**114.**	*Proteus mirabilis*	Negative	Negative	Negative	Negative	ND	ND

ND = Not Done
*Flagged as possible ESBLs by BDXpert, Confirmed by manual susceptibility method as an ESBL producer
Inoculum source is pellet from positive blood cultures for NG-Test CTX-M and NG-Test Carba 5
Inoculum source is isolated colonies from culture for BD Phoenix tests and Cepheid Carba-R PCR
Inoculum source is aliquot from the positive blood cultures for Easyplex SuperBug CRE Result

Created with
PDFBear.com
